# Supplementary figures and images for: Encoding time in neural dynamic regimes with distinct computational tradeoffs
Source: PLoS Comput Biol. 2022 Mar 3;18(3):e1009271. doi: 10.1371/journal.pcbi.1009271 (PMC8893702; doi:10.1371/journal.pcbi.1009271)

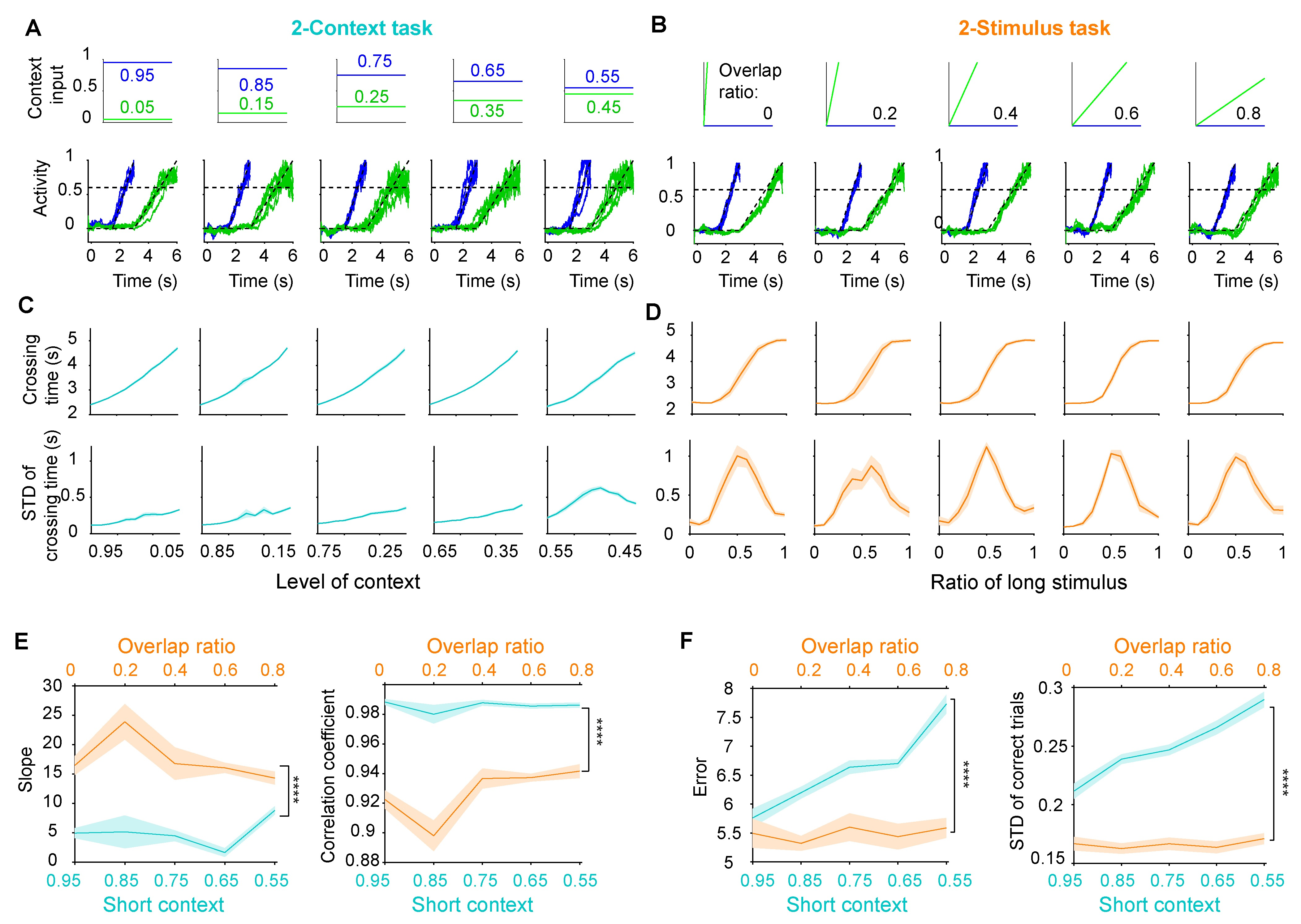

Supplement: S1 Fig — (A) Training on the 2-Context task with different analog context level pairs to signal the short (blue) and long (green) intervals (top), produced similarly timed short (blue) and long (green) intervals (bottom). Dashed lines denote the threshold used to measure the crossing time. (B) Training on 2-Stimulus task across different levels of overlap between the two input weight vectors (overlap ratio), quantified by the angle between the two weight vectors (top), and the corresponding learned output traces for short (blue) and long (green) intervals (bottom). (C) The mean (top) and standard deviations (bottom) of the crossing times across 10 simulations for the generalization experiments corresponding to the five conditions as in (A). (D) same as (C) but for 2-Stimulus task. (E) The sigmoid fit slopes of the generalization experiments in the five conditions of 2-Context task (cyan, as in A) were significantly lower than that for 2-Stimulus task (orange, as in B): two-way ANOVA, F1,90 = 123.1, P < 0.0001 (Left). The absolute correlation coefficients of the generalization experiments in the five conditions for 2-Context task (cyan, as in A) were significantly higher than that of the 2-Stimulus task (orange, as in B): two-way ANOVA on the Fisher-transformed data, F1,90 = 374.2, P < 0.0001 (right). (F) The mean error across all tested levels of the noise perturbation experiments in the five conditions for 2-Context task (cyan, as in A) is significantly higher than that for 2-Stimulus task (orange, as in B): two-way ANOVA, F1,90 = 106.1, P < 0.0001 (Left). Right, the same as the left but for standard deviations of the crossing times: two-way ANOVA, F1,90 = 625.7, P < 0.0001. (TIF) [file pcbi.1009271.s001.tif]

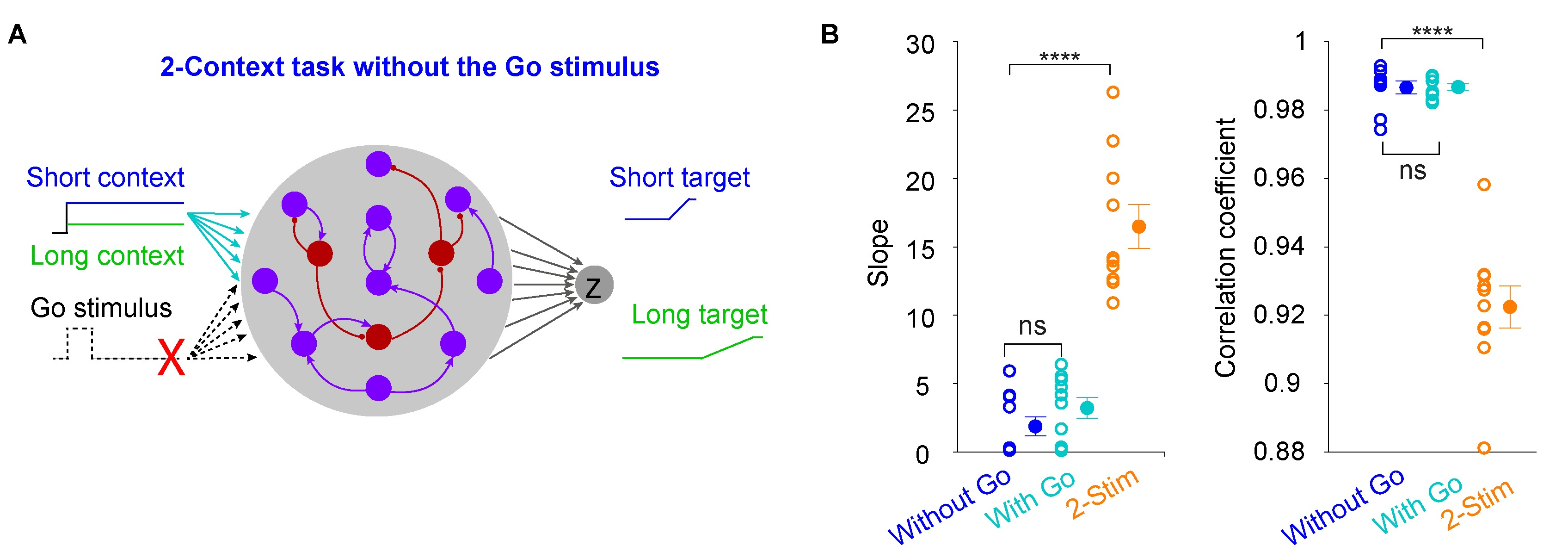

Supplement: S2 Fig — (A) Schematic of the 2-Context task without the Go stimulus. (B) Left, the sigmoid fit slopes in the generalization experiments for the 2-Context task without Go stimulus are not significantly different from the original 2-Context task, and still significantly lower than that for the standard 2-Stimulus task (one-way ANOVA with posthoc Tukey test, F2,27 = 53.4, ns: P = 0.669, ****: P < 0.0001). Right, the absolute correlation coefficients in the generalization experiments for the 2-Context task without Go stimulus not significantly different from the 2-Context task but significantly higher than that for the standard 2-Stimulus task (one-way ANOVA on the Fisher-transformed data with posthoc Tukey test, F2,27 = 112.9, ns: P = 0.957, ****: P < 0.0001). (TIF) [file pcbi.1009271.s002.tif]

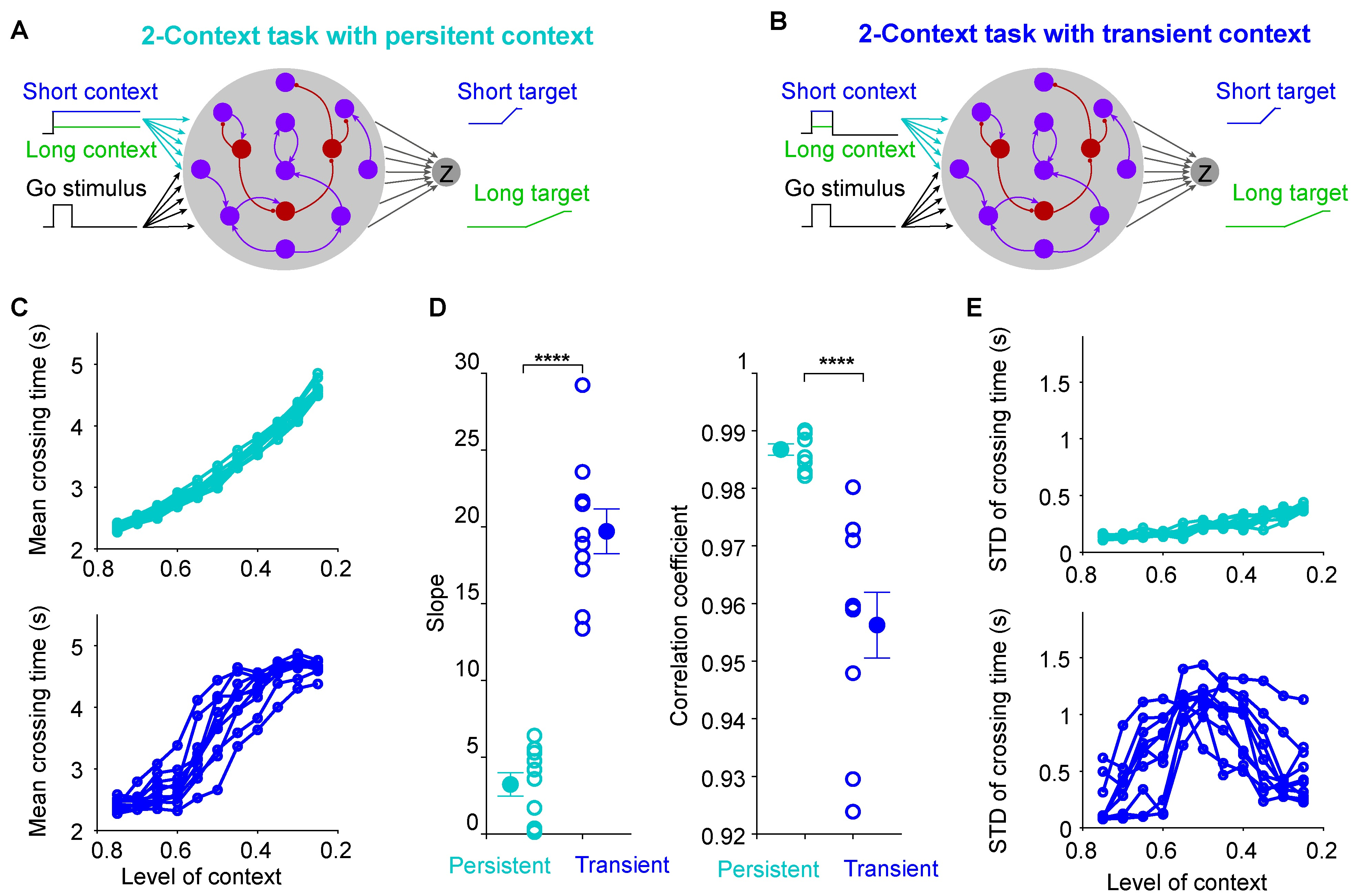

Supplement: S3 Fig — (A) Schematic of the standard 2-Cotnext task with persistent context input. (B) Schematic of a task in which the two intervals are signaled by the same brief input, but with different analog values. (C) Plots of the mean crossing time for each RNN across input conditions for the persistent (top) and transient (bottom) tasks. (D) Left, mean slope of the sigmoid fits for transient input task is significantly higher than that for the persistent 2-Context task (n = 10 simulations for each, two-sided t test, t18 = 9.98, P < 0.0001). Right, correlation coefficient between mean crossing times and input conditions for transient 2-Context is significantly lower than that for the persistent 2-Context task (n = 10 simulations for each, two-sided t test on Fisher-transformed values, t18 = 7.52, P < 0.0001). (E) Standard deviations of the crossing times for each RNN in the persistent 2-Context (top) and transient 2-Context (bottom) tasks, as a function of input conditions. (TIF) [file pcbi.1009271.s003.tif]

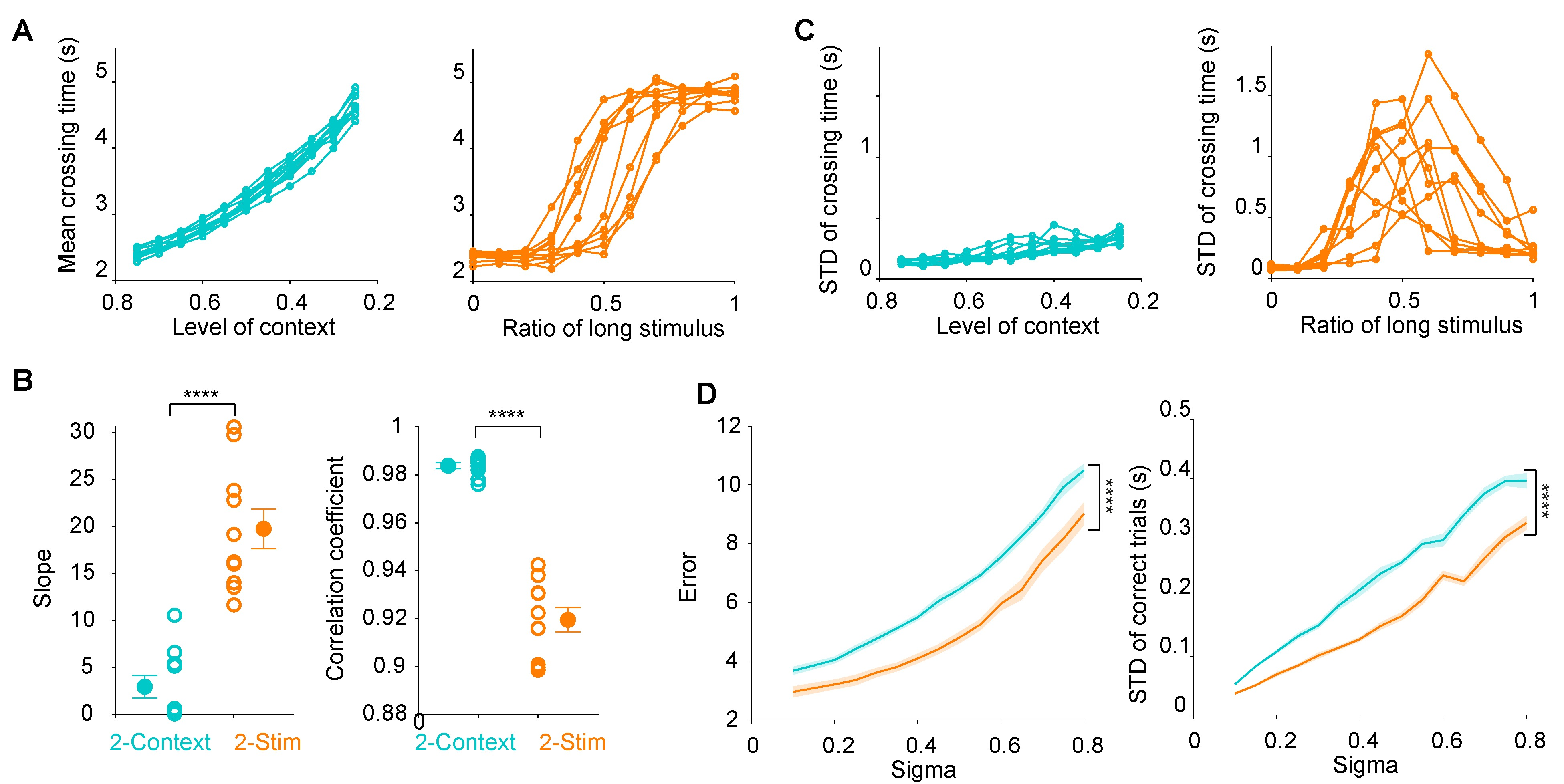

Supplement: S4 Fig — (A) Plots of the mean crossing time for each RNN across input conditions for the 2-Context (top) and 2-Stimulus (bottom) tasks. Insets, examples of the sigmoid-function fits for a single RNN (black). (B) Left, mean slope of the sigmoid fits for 2-Stimulus task is significantly higher than that for the 2-Context task (n = 20 simulations for each, two-sided t test, t18 = 6.91, P < 0.0001). Right, correlation coefficient between mean crossing times and input conditions for 2-Context task is significantly higher than that for the 2-Stimulus task (n = 20 simulations for each, two-sided t test on Fisher-transformed values, t18 = 16.56, P < 0.0001). (C) Standard deviations of the crossing times for each RNN in the 2-Context (top) and 2-Stimulus (bottom) tasks, as a function of input conditions. (D) Left, mean error (across 50 trials) for 2-Context task (cyan) is higher than that for 2-Stimulus task (orange) (n = 10 simulations, two-way ANOVA with mixed-effect design, F1,18 = 32.48, P < 0.0001). Right, mean standard deviation of the time of threshold-crossing across all correct trials for 2-Context task (cyan) is higher than that for 2-Stimulus task (orange) (F1,18 = 128.50, P < 0.0001). Data are presented as mean ± SEM. (TIF) [file pcbi.1009271.s004.tif]

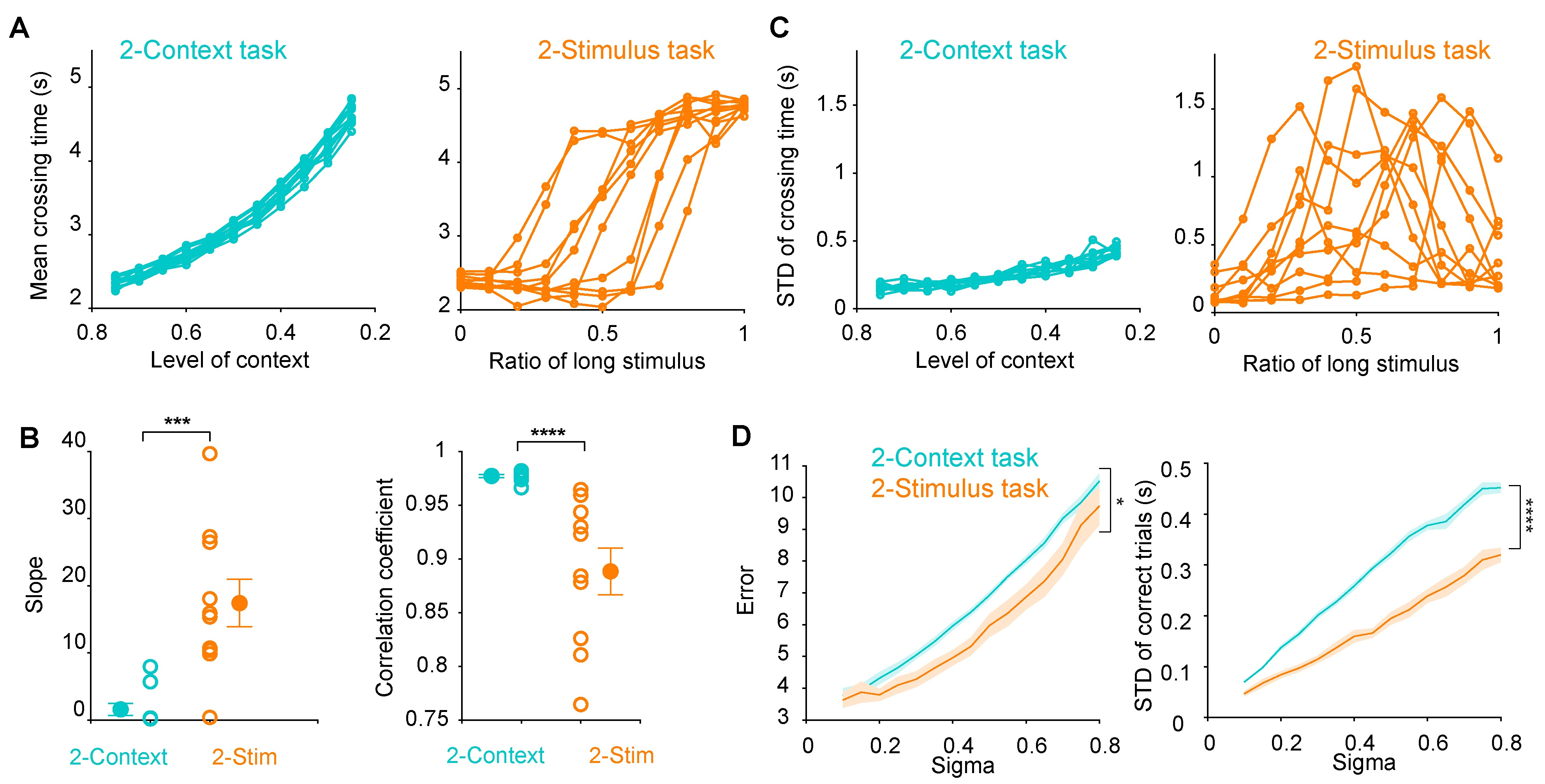

Supplement: S5 Fig — (A) Plots of the mean crossing time for each RNN across input conditions for the 2-Context (top) and 2-Stimulus (bottom) tasks. Insets, examples of the sigmoid-function fits for a single RNN (black). (B) Left, mean slope of the sigmoid fits for 2-Stimulus task is significantly higher than that for the 2-Context task (n = 20 simulations for each, two-sided t test, t18 = 4.35, P = 0.00039). Right, correlation coefficient between mean crossing times and input conditions for 2-Context task is significantly higher than that for the 2-Stimulus task (n = 20 simulations for each, two-sided t test on Fisher-transformed values, t18 = 6.48, P < 0.0001). (C) Standard deviations of the crossing times for each RNN in the 2-Context (top) and 2-Stimulus (bottom) tasks, as a function of input conditions. (D) Left, mean error (across 50 trials) for 2-Context task (cyan) is higher than that for 2-Stimulus task (orange) (n = 10 simulations, two-way ANOVA with mixed-effect design, F1,18 = 5.78, P = 0.027). Right, mean standard deviation of the time of threshold-crossing across all correct trials for 2-Context task (cyan) is higher than that for 2-Stimulus task (orange) (F1,18 = 86.03, P < 0.0001). Data are presented as mean ± SEM. (TIF) [file pcbi.1009271.s005.tif]

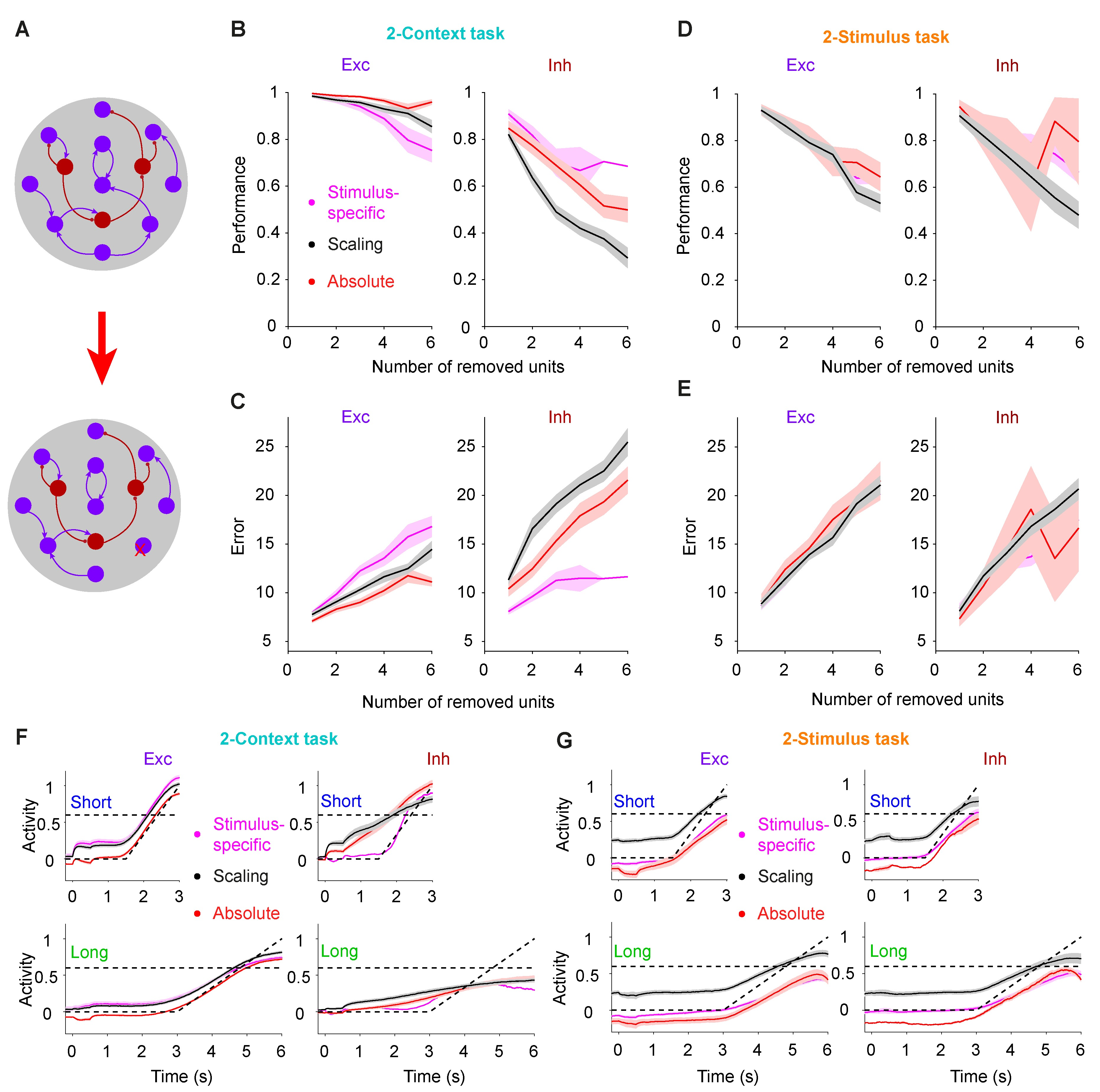

Supplement: S6 Fig — (A) Schematic of the deletion experiments. To delete a given unit denoted by the red arrow (bottom), all in and out weights of the recurrent weight matrix of that units were set to zero. (B) Performance of RNNs trained on the 2-Context task after progressively deleting units from specific temporal classes: stimulus-specific, scaling, and absolute temporal classes for both excitatory (left) and inhibitory (right) units. For each data point, units were randomly selected 10 times, and 10 test trials were obtained. A three-way ANOVA revealed highly significant effects of main temporal-class (F2,619 = 31, P < 10−12) and Ex-Inh (F2,619 = 390, P < 10−66) factors. Additionally, there was a highly significant interaction between temporal-class and Ex-Inh class (F2,619 = 27, P < 10−10) and multi-comparison analyses showed that performance for inhibitory scaling cells was significantly lower than all other 5 deletion manipulations (P < 0.0001 for all comparisons). (C) Similar to (B) but for error. As in (B), there were highly significant main effects (F2,619 = 34, P < 10−14, and F2,619 = 118, P < 10−24, for temporal-class and Ex-Inh, respectively), as well as a significant interaction between temporal-class and Ex-Inh (F2,619 = 46, P < 10−18). And again the inhibitory scaling cells increased the error more than all other deletion manipulations (P < 0.0001 for all comparisons). (D-E) There were no main effects of temporal-class or Ex-Inh that were consistently significant for both the performance and error measure. The interaction between temporal-class and Ex-Inh was either trending (F2,619 = 2.5, P = 0.08) or mildly significant (F2,619 = 3.6, P = 0.027) for the performance and error analyses, respectively. Data are presented as performance mean ± SEM across 20 RNNs. Notice that the performance of stimulus-specific units in (D) and (E) (magenta) are very similar to, and mostly obscured by the absolute traces (red). (F) Mean output traces across 20 simulations when deleting 6 e [file pcbi.1009271.s006.tif]

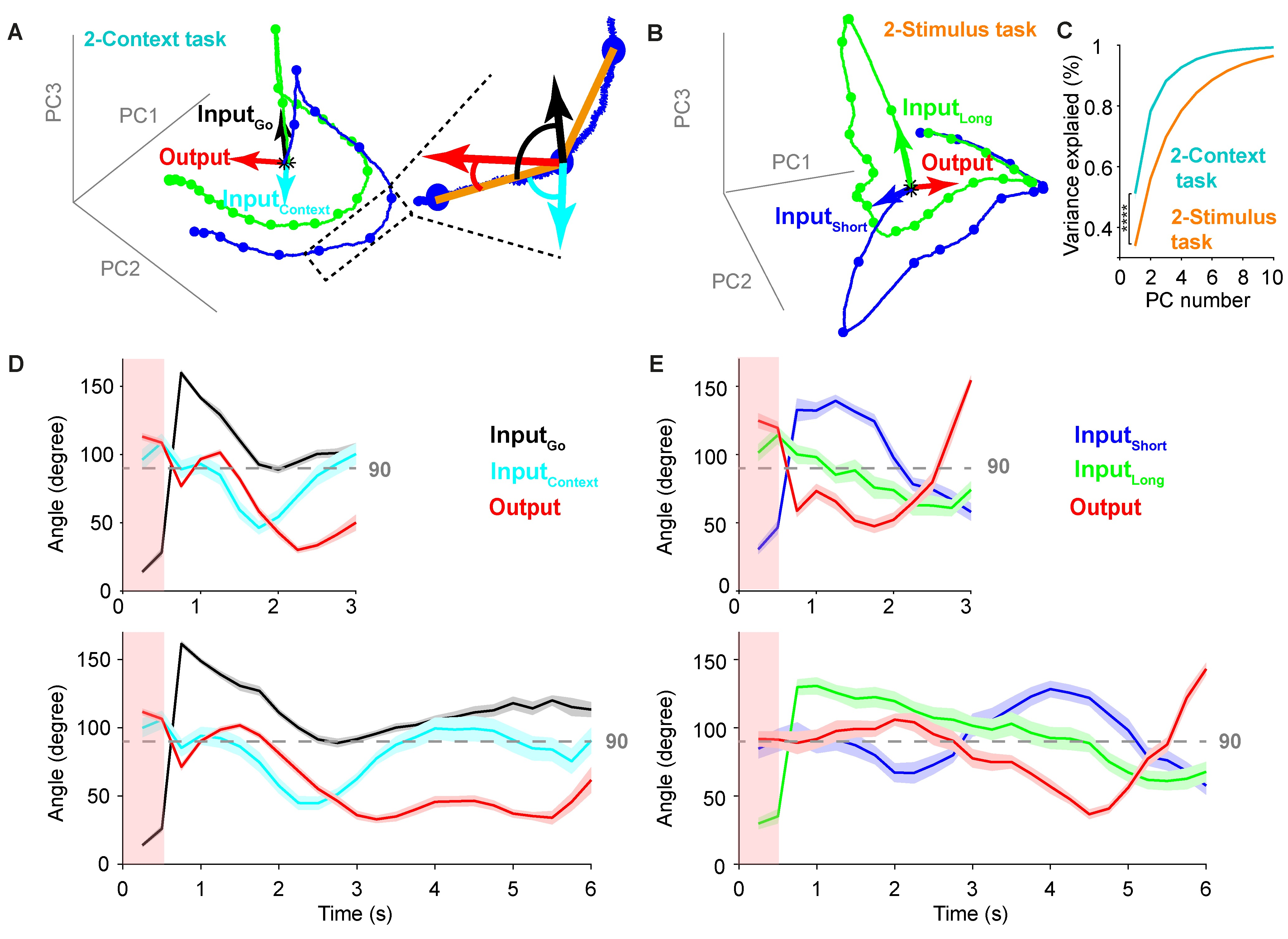

Supplement: S7 Fig — (A) For the 2-Context task, recurrent unit dynamics for the short (blue) and long (green) intervals were projected into the first three PC spaces. Asterisks denote the onset of inputs (t = 0), arrows denote the corresponding weights vectors (InputGo, black; InputContext, cyan; and Output, red) projected onto the same PC space. Color dots denote the 250 ms intervals along each trajectory. Inset, schematic of angles between segments of the approximate RNN trajectory (orange) and the three weight vectors. These vectors were used to compute the pairwise angles to the InputGo, InputContext and Output vectors. (B) Similar to (A) but for 2-Stimulus task, but here the two input vectors represented the InputShort (blue) and InputLong (green) weight vectors. (C) Same number of PCs explained more variance for 2-Context task than that for 2-Stimulus task (Two-way ANOVA, F(1, 38) = 255.6 and P < 0.0001). (D) Average pairwise angles between segments of short (top)/long (bottom) dynamics and inputs/output vectors as in (A) for 2-Context task (20 simulations, data presents as Mean ± SEM). Shaded area donted the duration of the transient InputGo (E) Same as in (D) but for 2-Stimulus task. The shaded area denotes the duration of the transient InputShort and InputLong. (TIF) [file pcbi.1009271.s007.tif]

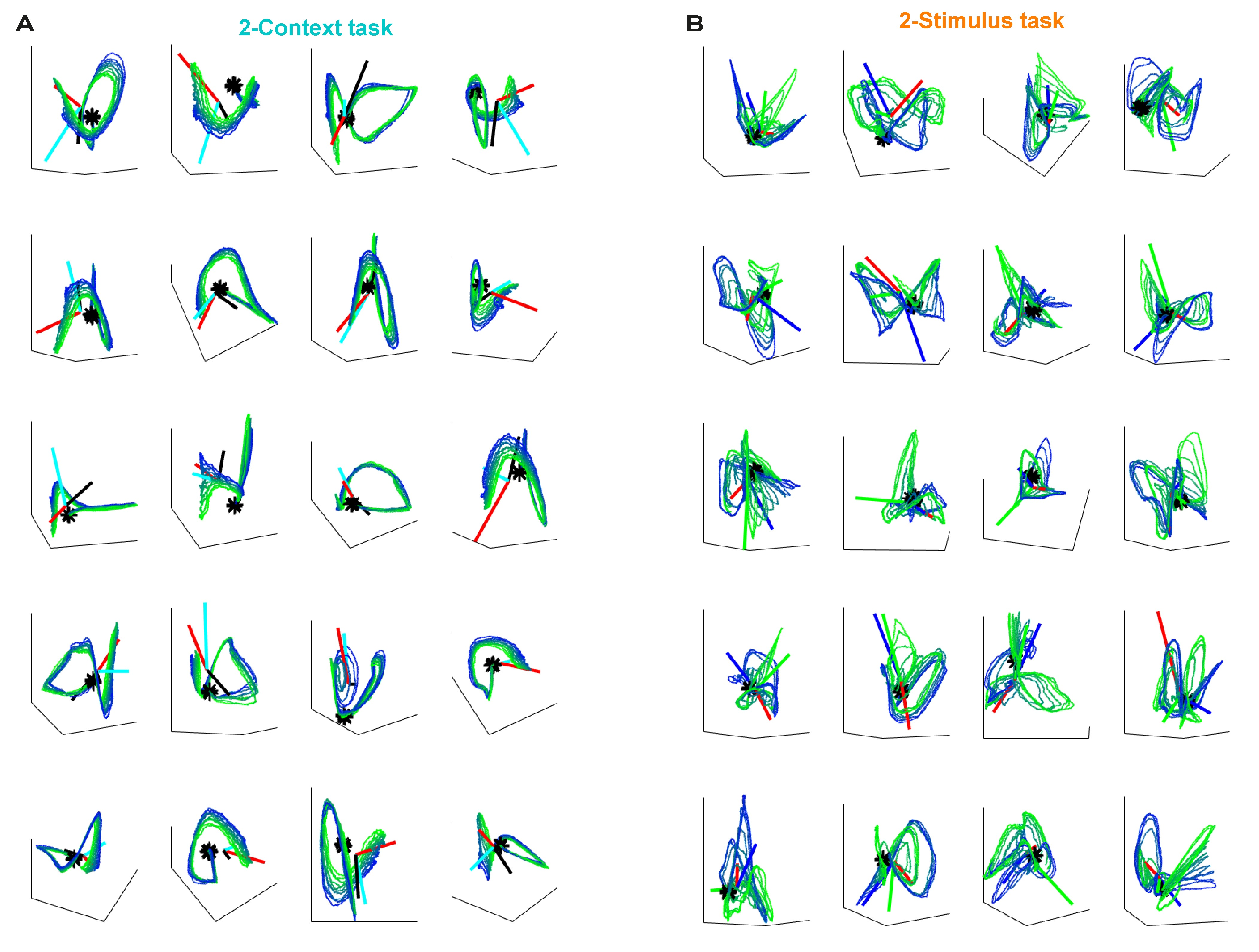

Supplement: S8 Fig — (A) Recurrent dynamics corresponding to different context levels (denoted by the color) as in Fig 2 were projected into the first three PCs in 20 RNNS trained on 2-Context task. The arrows denoted the directions of InputGo (black), InputContext (cyan), and Output (red) weights projected into the same PC space. (B) similar as in (A) but for the 2-Stimulus task. (TIF) [file pcbi.1009271.s008.tif]
